# Anti-Tuberculous Activity of Treponemycin Produced by a *Streptomyces* Strain MS-6-6 Isolated from Saudi Arabia

**DOI:** 10.3390/molecules20022576

**Published:** 2015-02-02

**Authors:** Mahmoud A. Yassien, Hossam M. Abdallah, Ali M. El-Halawany, Asif A. M. Jiman-Fatani

**Affiliations:** 1 Department of Natural Products and Alternative Medicine, Faculty of Pharmacy, King Abdulaziz University, P.O. Box 80260, Jeddah 21589, Saudi Arabia; E-Mails: hmafifi2013@gmail.com (H.M.A.); ahalawany2003@yahoo.com (A.M.E.-H.); 2 Department of Microbiology and Immunology, Faculty of Pharmacy, Ain-Shams University, Cairo, Egypt; 3 Department of Pharmacognosy, Faculty of Pharmacy, Cairo University, Cairo 11562, Egypt; 4 Department of Medical Microbiology and Parasitology, Faculty of Medicine, King Abdulaziz University, P.O. Box 80260, Jeddah 21589, Saudi Arabia.; E-Mail: afatani@kau.edu.sa

**Keywords:** *Streptomyces mutabilis*, treponemycin, anti-tuberculous activity

## Abstract

A *Streptomyces* strain MS-6-6 with promising anti-tuberculous activity was isolated from soil samples in Saudi Arabia. The nucleotide sequence of its 16S rRNA gene (1426 bp) evidenced a 100% similarity to *Streptomyces mutabilis*. Through an anti-tuberculous activity-guided approach, a polyketide macrolide was isolated and identified as treponemycin (TP). The structure of the isolated compound was determined by comprehensive analyses of its 1D and 2D NMR as well as HRESI-MS. In addition to the promising anti-tuberculous activity (MIC = 13.3 µg/mL), TP showed broad spectrum of activity against the Gram positive, Gram negative strains, and *Candida albicans*. Improvement of TP productivity (150%) was achieved through modification in liquid starch nitrate medium by replacing KNO_3_ with corn steep liquor and yeast extract or tryptone, and removing CaCO_3_ and K_2_HPO_4_. The follow up of TP percentage as well as its metabolites profile for each media was assessed by LC/DAD/MS.

## 1. Introduction

Tuberculosis (TB) is one of the most serious infectious diseases. One-third of the world’s population is infected with *Mycobacterium tuberculosis*. One of the most significant challenges in treating TB patients is the development of microbial resistance to many of the currently used anti-TB drugs [1].

According to the WHO estimates, the incidence of all forms of TB and its related complications was increased by 6.2% between 1990 and 2004. In Saudi Arabia, the highest level of TB infection was reported in Jeddah (31.1%) followed by Riyadh (23.2%) [2]. Accordingly, there is great interest in obtaining novel antibiotics with promising anti-tuberculous activity.

The genus *Streptomyces*, filamentous soil bacteria, have been described as the greatest source of the commercially available antibiotics [3,4,5]. *Streptomyces* are also reported to produce other valuable bioactive secondary metabolites acting as antitumor agents, immunosuppressive agents and enzymes [6,7,8]. Different types of anti-tuberculous antibiotics are produced by *Streptomyces* sp. The first one is streptomycin, discovered in 1943. Other anti-tuberculous antibiotics produced by *Streptomyces* include d-cycloserine and kendomycin that produced from *S. garyphalus* and *S. violaceoruber*, respectively [9,10]. Moreover, the microbial production of secondary metabolites including antibiotics is affected greatly by nutritional condition [11]. The medium supplies nutrients for growth, energy, building of the cell substances and biosynthesis of the desired fermentation products. The sources of carbon and nitrogen in the medium are of particular importance, since microbial cells and fermentation products are composed largely of these two elements [12]. Therefore, the composition of the fermentation medium should be taken in consideration during establishment of the suitable fermentation experiments to maximize the production of secondary metabolites.

This work describes the genetic identification of *Streptomyces* strain(s) with promising anti-tuberculosis activity collected from Saudi Arabia soil, purification and structure determination of the bioactive agent, evaluation of the antimicrobial activity of the purified compound, and improvement of bioactive compound productivity.

## 2. Results and Discussion

### 2.1. Screening of Anti-Tuberculous Activity of Streptomyces Isolates

A total of 492 *Streptomyces* isolates were obtained from 93 soil samples collected from different localities in the Western Region of Saudi Arabia. The anti-tuberculous activity of the isolates was evaluated against *M. tuberculosis* (ATCC 25177) by cup-diffusion techniques. The results showed that 155 of the tested isolates have different degrees of anti-tuberculous activity (Supplementary Table S1) from which eight isolates have promising anti-tuberculous activity (Table 1). These isolates were assessed for their cytotoxicty against normal human fetal lung fibroblast cell line (MRC-5). The obtained results (Table 1) showed that all the tested *Streptomyces* extracts have weak cytotoxic effects (IC_50_ ≥ 240 µg/mL). Accordingly, the isolate coded MS-6-6 with the strongest anti-tuberculous activity was selected for further studies.

**Table 1:** The anti-tuberculous activity of the selected *Streptomyces* extracts and their cytotoxic activities against normal human fetal lung fibroblast cell line (MRC-5).

Strain Number	Inhibition Zone (mm)	Equivalent Activity to Rifampicin (mg/L)	Cytotoxic Activity (IC_50_) (µg/mL)
MS-6-6	29	0.08	280
MS-18-4	20	0.03	315
MKS-9-3	20	0.03	275
JS-8-4	20	0.03	315
JS-9-4	24	0.05	265
JS-22-9	27	0.06	240
GS-42-1	24	0.05	320
ES-15-3	22	0.04	260

### 2.2. Identification of Streptomyces Strain MS-6-6

**Figure 1:**
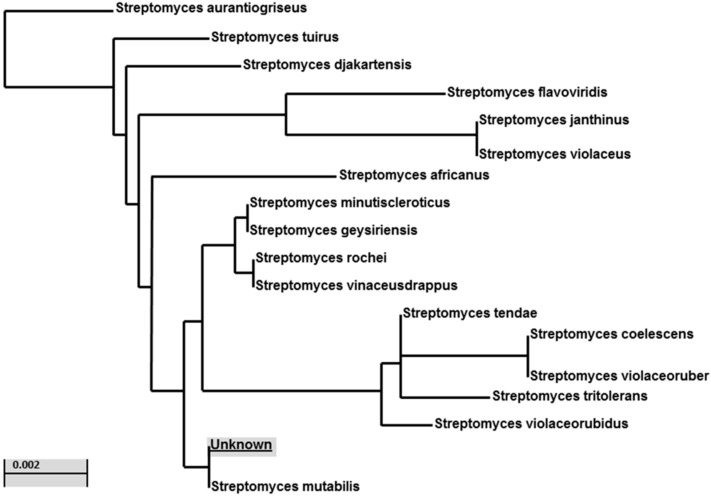
Phylogenic relationships between *Streptomyces* strain MS-6-6 and related species based on partial nucleotide sequence (1426 bp) of the 16S rRNA gene. The tree was constructed using the neighbor-joining method. The scale bar indicates a 0.002 substitution per nucleotide position.

According to the cultural, physiological, and microscopical properties of the selected isolate MS-6-6, it was identified as a member of the genus *Streptomyces* (Supplementary Figures S1 and S2, Tables S2–S4). The nucleotide sequence of the 16S rRNA gene (1426 bp), obtained from the MS-6-6 isolate was aligned with all presently available 16S rRNA gene sequences in the GeneBank databases. The results showed high similarity (98%–100%) to the *Streptomyces* 16S rRNA genes (Accession no. NR 044139.1). Phylogenetic analysis using the 16S DNA gene sequence suggests the strain is similar to *Streptomyces mutabilis* (Figure 1).

### 2.3. Isolation and Structure Elucidation of the Bioactive Compound from the Streptomyces Mutabilis

The antimicrobial activity of the fraction solutions was evaluated and the results revealed that fraction A-3 showed promising antimicrobial activity against the tested Gram-positive, Gram-negative bacteria, *C. albicans* and *M. tuberculosis* (zones of inhibition ranged between 15 and 24 mm), while A-2 fraction exhibited a weak antimicrobial activity (range of inhibition zones 11–15 mm). However, no antimicrobial activity was obtained by testing fraction A-1 (Supplementary Table S5).

Chromatographic purification of fraction A-3 resulted in the isolation of one compound MYA-3 in pure form as a white amorphous powder (4.8 mg). The molecular formula was deduced to be C_28_H_43_NO_6_ from HRESI-MS which displayed a molecular ion peak at *m/z* 488.2874 for [M−H]^−^ in negative ion mode and a molecular ion peak at *m/z* 512.3406 for [M+Na]^+^ in positive mode. ^1^H-NMR spectra of MY-A3 displayed four doublet methyl protons (δ_H_ 0.80, 0.84, 0.85, 1.03, *J* = 6 Hz) for methyls attached to C_5_, C_4_, C_8_, and C_10_ respectively, three deshielded protons (δ_H_ 3.86 *m*, 4.10 d, *J* = 9.6 Hz, and 5.0, *m*) for H_3_, H_11_ and H_17_ respectively (Table 2). Moreover, three olefinic protons (δ_H_ 6.20 *m*, 6.40 *m*, and 6.80 *d*, *J* = 10) corresponding to H_15_, H_14_ and H_13_, respectively, were observed. Furthermore, ^13^C-NMR and DEPT spectra revealed the presence of 28 carbons; four methyls, eight methylenes, 12 methane and four quaternary carbons. The spectrum displayed two characteristic peaks at 116.1 and 176.1 for α,β-unsaturated nitrile and carboxylic carbons, respectively. Complete correlations between carbons and protons were achieved through two-dimensional NMR spectra, including, COSY, HSQC, HMBC and NOESY (Supplementary Figures S3–S7). All spectroscopic data proved that compound MYA-3 belongs to the polyketide macrolide group and identified it as treponemycin (TP) (borrelidin) [13,14,15,16,17,18,19,20]. Although, TP was first isolated from *Streptomyces rochei*, it was subsequently identified in other *Streptomyces* species such as *S. californicus*, *S. rimosus*, and *S. heilongjiangensis* [16,17,18,19], but this is the first time production of TP by *S. mutabilis* has been reported.

**Table 2:** ^1^H- and ^13^C-NMR, COSY and HMBC spectral data of compound (**MYA-3**) (CDCl_3_).

Position	H	C	COSY	HMBC (H with C)
1	-	172.1	-	-
2	2.42, 2.32 *m*	39.3(CH_2_)	3.86	172.1, 69.9, 35.1
3	3.86, *m*	69.9(CH)	2.32, 2.42	43, 172.1
4	1.67, m	35.1(CH)	3.86, 1.25, 0.84	69.9, 39.3, 27.2
5	0.92, 1.25	43.1(CH_2_)	1.67, 1.57	18.23, 47.8, 69.9
6	1.57	27.2(CH)	1.10, 0.97, 1.25	26.3, 35.1
7	0.97, 1.10	47.8	1.62, 1.57	37.5, 43.1
8	1.62	26.3(CH)	0.85, 1.03, 1.1	65.3, 27.2
9	1.03, 0.76	37.5(CH_2_)	1.85, 1.62	14.9, 73.2, 26.3
10	1.85	65.3(CH)	4.1, 1.03	118.1, 26.3
11	4.10, *d*, *J* = 9.6	73.2(CH)	1.85	116.1, 37.47, 143.9, 14.9
12	-	118.1	-	-
13	6.80, *d*, *J* = 10.8	143.9(CH)	6.40	118.1, 138.5, 73.2,126.9
14	6.40, *m*	126.9(CH)	6.20, 6.80	116.1, 35.9, 143.9
15	6.20, *m*	138.5(CH)	6.40, 2.56	143.9, 35.9, 76.6
16	2.56, 2.59, *m*	35.9(CH_2_)	5.0, 6.20	126.9, 138.5, 76.5, 45.9
17	5.0, *m*	76.5 (CH)	2.56, 2.59, 2.70	172.1,138.5, 29.6,35.9
18	2.70, *t*	45.9 (CH)	1.30, 1.97, 2.51	176.1, 76.5, 47.8, 29.6
19	1.97, 1.30*m*	29.6(CH_2_)	2.70, 5.0, 2.51	76.5, 47.8, 31.1, 25.2
20	1.83, 1.79	25.2 (CH_2_)	1.30, 2.03, 1.90	45.9, 47.8
21	2.03, 1.90 *m*	31.1(CH_2_)	1.83, 1.79, 2.51	176.1, 45.9, 47.8
22	2.51, *m*	47.8(CH)	1.90, 2.03, 2.70	176.1, 76.5, 45.9, 29.6, 31.1
CH_3_ at C_4_	0.84, *d*, *J* = 6	17.0	-	35.1, 43.1, 69.9
CH_3_ at C_6_	0.80, *d*, *J* = 6	18.2	-	27.2, 47.8, 43.1
CH_3_ at C_8_	0.85, *d*, *J* = 6	20.2	-	26.3, 37.5, 47.8
CH_3_ at C_10_	1.04, *d*, *J* = 6	14.9	-	65.3, 73.2
CN	-	116.1	-	-
COOH	-	176.1	-	-

### 2.4. Antimicrobial Activity of Treponemycin

The results showed that TP has a strong antimicrobial activity with the MICs ranges of 1.7–16.7 and 8.3–26.7 µg/mL against the tested Gram-positive, and Gram-negative strains, respectively. In addition, it has promising activity against *M. tuberculosis* ATCC 25177 and *C. albicans* with MIC values of 4.15 and 13.3 µg/mL, respectively (Table 3). Previous biological screening indicated that TP exhibits antiviral [20] and antibacterial activities [21], which presumably arise from its inhibition of threonyl-tRNA synthetase and protein synthesis [22]. It also displays potent antimitotic properties at low concentrations due to inhibition of cyclindependent kinase. None of the previous studies observed any anti-tuberculous activity of TP.

**Table 3:** Antimicrobial activity of the purified antimicrobial agent, treponemycin, produced by *Streptomyces* isolate MS-6-6.

Organisms	MIC (µg/mL)
*Mycobacterium tuberculosis*	4.17
*Staphylococcus epidermidis*	1.7
*Streptococcus pyogenes*	2.1
*Bacillus subtilis*	2.9
*Escherichia coli*	8.3
*Clostridium perfringens*	16.7
*Brucella melitensis*	16.7
*Pseudomonas aeruginosa*	26.7
*Proteus mirabilis*	11.3
*Candida albicans*	13.3

MIC is the mean of three repeated experiments.

### 2.5. Improvement of Treponemycin Productivity

#### 2.5.1. Improvement of Antimicrobial Activity

Due to the importance of TP and its interesting biological activities, many attempts have been made to prepare it through chemical synthesis. The enantioselective total synthesis of TP is highly complicated and expensive, consequently a simpler method is vital [13,21,23]. *S. mutabilis*, isolated in the present study, has the ability to produce promising levels of TP during the fermentation process. In addition, the present study established a suitable system for purification of TP from the fermentation supernatant. In industrial microbiology, more that 70% of the produced antibiotics depend on *Streptomyces* spp. [5]. Therefore, from an economic view it is more interesting to depend on the fermentation of *S. mutabilis* for microbial production of TP.

The composition of the fermentation medium is of principal importance in the design of successful laboratory experiments [11]. Therefore, improvement of the microbial production of TP was performed through nutritional modification by changing the growth media and detection of the level of TP production through measuring the antimicrobial activity of growth supernatant. Accordingly, different fermentation media (Table 4) were tested for their suitability to improve the TP productivity of *S. mutabilis*.

**Table 4:** Ingredients of different fermentation media.

Ingredients (g/L)	M1 *	M2	M3	M4	M5	M6	M7	M8
**CSL**	-	-	20.0	20.0	20.0	10.0	10.0	20.0
**Yeast extract**	-	10.0	10.0	-	-	10.0	-	-
**Peptone**	-	-	-	10.0	-	-	-	-
**Tryptone**	-	10.0	-		10.0	-	10.0	-
**Soluble starch**	20.0	10.0	20.0	20.0	20.0	-	-	10.0
**Glucose**	-	10.0	-	-	-	20.0	10.0	10.0
**KNO_3_**	2.0	-	-	-	-	-	-	-
**CaCO_3_**	3.0	-	-	-	-	-	-	-
**MgSO_4_ 7H_2_O**	0.5	0.5	0.5	0.5	0.5	0.5	0.5	0.5
**NaCl**	0.5	0.5	0.5	0.5	0.5	5.0	5.0	5.0
**K_2_HPO_4_**	1.0	-	-	-	-	-	-	-
**FeSO_4_ 7H_2_O**	0.01	0.01	0.01	0.01	0.01	-	-	-
**MnCl_2_**	0.01	0.01	0.01	0.01	0.01	-	-	-
**ZnSO_4_ 7H_2_O**	0.01	0.01	0.01	0.01	0.01	-	-	-

M1 * = Liquid starch nitrate medium; CSL = Corn steep liquor.

The results (Table 5) revealed that the maximum antimicrobial activity of *S. mutabilis* was obtained by using M3 and M5 media, whereby the size of the inhibition zones was increased by approximately 1.2–1.35 fold more than that produced by using the original medium (M1, liquid starch nitrate medium). M3 and M5 are modified forms of the M1 medium made by replacing KNO_3_ with corn steep liquor (CSL) and yeast extract or tryptone, and removing CaCO_3_ and K_2_HPO_4_. The use of organic nitrogen sources, such as CSL and yeast extract or tryptone, is much better than inorganic nitrogen source, as KNO_3_, for microbial production of secondary metabolites as due to their complex nature, organic nitrogenous compounds act as a slow release nitrogen sources which play important roles in the high productivity of secondary metabolites such as antibiotics. The suitability of using organic compounds as nitrogen sources for antibiotic production was reported by other investigators [24].

**Table 5:** TP titer and antimicrobial activity of *S. mutabilis* supernatant using different fermentation media.

Inhibition Zones (mm)					TP Titer
Media	*M. tuberculosis*	*S. epidermisis.*	*E. coli*	*C. albicans.*	mg/L
**M1**	24	18	16	14	0.45
**M2**	17	15	14	12	0.38
**M3**	29	24	21	18	0.67
**M4**	26	21	18	15	0.50
**M5**	28	22	19	16	0.68
**M6**	17	15	14	12	0.40
**M7**	17	15	14	11	0.36
**M8**	22	17	15	13	0.45

#### 2.5.2. LC-DAD/ESI-MS Analysis of TP and Its Metabolites in Different Culture Media

**Figure 2:**
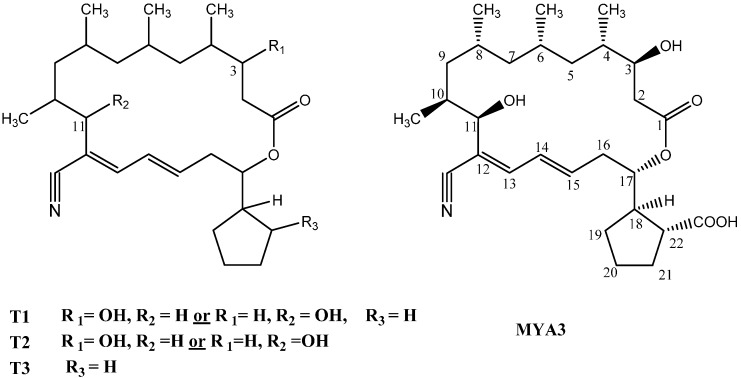
Structure of trepenomycin (MYA-3) and its metabolites (T1–T3).

**Figure 3:**
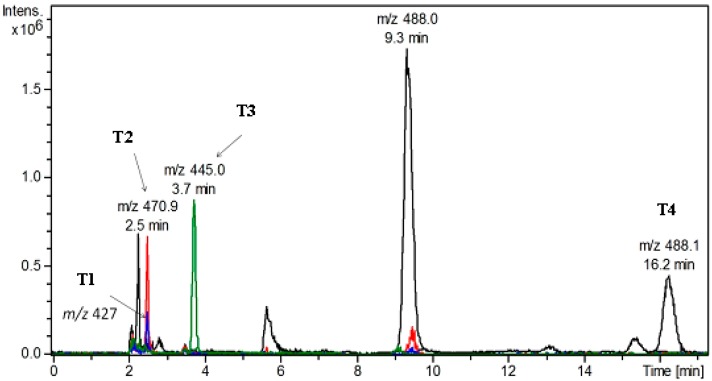
Representative (overlay) of extracted ion chromatograms (EIC) of treponemycin and its metabolites in different culture media.

The fermentation supernatants of the modified media that showed promising antimicrobial activity were selected to determine the titers of TP on the basis of LC-DAD/MS analysis calibrated with an authentic TP standard. All the tested media caused production of TP (*m/z* 488.1 at 9.3 min) with different concentrations (Table 5) in addition to other metabolites (T1–T4) (Figure 2 and Figure 3). The highest titer of TP was produced by using M3 and M5 media, in which TP was the major metabolite with approximate concentration range 0.67–0.68 mg/L. Accordingly, the use of M3 and M5 media increased the TP titer by approximately 1.5 fold as compared to that of the original medium (M1).

#### 2.5.3. Identification of Metabolites

Metabolite profiling of the tested media revealed the presence of TP in addition to four major related metabolites (T1–T4) at *m/z* 426 [M−COOH−H_2_O]^−^, *m/z* 471 [M−H_2_O]^−^, *m/z* 445 [M−CO_2_]^−^ radicle ion, and *m/z* 488 [Iso-TP]^−^, respectively.

**Figure 4:**
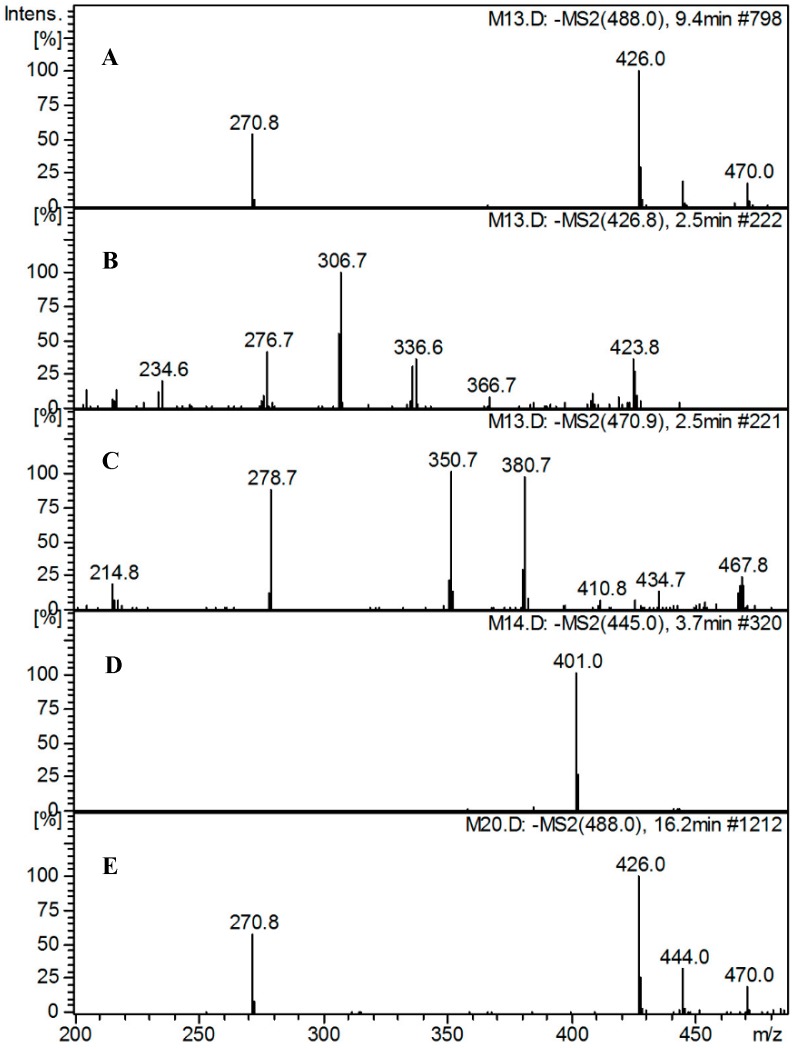
Mass spectra of compound MYA-3 and its detected metabolites. (**A**) MS2 spectra of compound MYA-3; (**B**) MS2 spectra of T1; (**C**) MS2 spectra of T2; (**D**) MS2 spectra of metabolite T3; (**E**) MS2 spectra of metabolite T4.

T1 showed a molecular ion peak at *m/z* 426 indicating the loss of carboxylic group (45 u) and hydroxyl moiety (17 u) (either from position 3 or 11) (Figure 4). T2 with a molecular ion peak at *m/z* 471 was identified as dehydroxytrepenomycin (due to loss of a hydroxyl group from position 3 or 11). Moreover, T3 showed a molecular ion peak at 445 indicating the loss of a carboxylic acid group. Finally T4 could be tentatively identified as an isomer of TP as it shows the same molecular ion at *m/z* 488 (Figure 4).

Metabolites T1 and T2 were produced in M3 medium, metabolite T3 was produced in M4 and M5 media, while metabolite T4 was produced in M2 and M6–M8 media. Moreover, media M2, M6, M7 showed variable concentrations of metabolite T2. It was reported that nitrile, lactone and probably the hydroxyl functions are essential for the antimicrobial activity of TP [25]. Therefore, the increase in antibacterial activity due to media change could be mainly due to the increase of TP concentration, as the other metabolites (T1–T4) are lacking the functional groups necessary for activity.

## 3. Experimental Section

### 3.1. Microorganisms

Mycobacterium tuberculosis (ATCC 25177), Staphylococcus epidermidis (ATCC 12228), Streptococcus pyogenes (ATCC 19615), Bacillus subtilis (ATCC 6633), Escherichia coli (ATCC 25922), Pseudomonas aeruginosa (ATCC 27853), Proteus mirabilis (ATCC 14153), Brucella melitensis 16M (ATCC 23456), Clostridium perfringens (ATCC 19404), and Candida albicans (ATCC 10231) were used. All test standard strains, except Candida albicans and Mycobacterium tuberculosis, were maintained onto Mueller Hinton Agar slants. C. albicans was maintained on Sabouraud dextrose agar slants, while M. tuberculosis was maintained on Lowenstein Jensen medium and Middlebrooks 7H9 agar medium. Standard strains, except M. tuberculosis, were stored in skimmed milk or 15% glycerol in Brain Heart Infusion at −86 °C.

### 3.2. Isolation of Streptomyces Strains

Isolation of *Streptomyces* was carried out as described by Jaradat *et al*. [26]. In brief, soil suspensions (1%) were prepared and plated onto the surface of starch-nitrate agar medium (20 g soluble starch, 2.0 g KNO_3_, 1.0 g K_2_HPO_4_, 0.5 g MgSO_4_, 0.5 g NaCl, 3.0 g CaCO_3_, 0.01 g FeSO_4_, 0.01 g MnCl_2_, 0.01 g ZnSo_4_, 20 g agar per liter) and incubated at 30 °C. After 5 to 7 days of incubation, colonies having the typical culture characteristics of *Streptomyces* (leathery or fibrous colonies appearing as granular powders with hard texture) were picked and purified by streak-plate technique on the same medium. Purified isolates were maintained onto starch-nitrate agar slants at 4 °C and subcultured every month.

### 3.3. Evaluation of Antimicrobial Activity

#### 3.3.1. Fermentation and Preparation of *Streptomyces* Clear Supernatants

Colonies from 4- to 5-days old starch nitrate cultures were suspended in normal saline solution and appropriately diluted to give a final count of 1−2 × 10^5^ CFU/mL determined by viable count technique. Aliquots (0.5 mL) of *Streptomyces* spore suspensions (1–5 × 10^5^ CFU/mL) were used to inoculate liquid starch nitrate medium (50 mL) in 250 mL Erlenmeyer flasks and incubated in an rotary shaking-incubator (S19R-2, Sheldon Manufacturing Inc., Cornelius, OR, USA) at 200 rpm and 30 °C for 96 h. After incubation, a known volume of culture broth was centrifuged at 6000× *g* for 20 min to obtain a clear supernatant and the cell pellet was collected to determine the dry cell weight.

#### 3.3.2. Bacterial and Fungal Susceptibility Testing

Antimicrobial susceptibility testing was done according to the standardized cup-diffusion technique [27]. In brief, colonies from overnight cultures of the tested standard strains were suspended in saline solution and appropriately diluted to match the turbidity of standard 0.5 on McFarland scale, diluted in saline (1:100) and used for seed inoculation of 25 mL agar plates (Mueller-Hinton agar for bacterial strains or Sabouraud dextrose agar for *C. albicans*) to the final count of 2–5 × 10^5^ CFU/mL as determined by viable count technique. Aliquots (200 μL) of the tested samples (*Streptomyces* clear supernatant, the prepared fractions from the lyophilized supernatant or the bioactive compound) were evaluated by as described. Each experiment was carried out in triplicate and mean value was calculated. Ciprofloxacin (for antibacterial activity) and fluconazole (for antifungal activity) were used as positive control.

#### 3.3.3. Evaluation of Anti-Tuberculous Activity

##### Preparation of Bacterial Suspension

Colonies from 14 to 21 days old culture of *M. tuberculosis* (ATCC 25177) were transferred into sterile glass vial containing 2.5 mL of Middlebrooks 7H9 fresh broth with glass beads. After vortexing for 1 min and waiting for 15 min, the supernatant was transferred to another tube and diluted with the same medium to match the turbidity of McFarland 1.0 standard (equivalent to 3 × 10^8^ CFU/mL). The final suspension was diluted 1:20 of broth and used for inoculation of assay plates.

##### Anti-Tuberculous Activity by Cup-Diffusion Techniques

Aliquots (100 μL) of the prepared *M. tuberculosis* suspension were used for surface inoculation of a Middlebrooks 7H10 agar medium (Difco, Detroit, MI, USA) supplemented with 10% OADC (oleic acid-albumin-dextrose-catalase). Aliquots (200 μL) of the tested samples were dripped in the prepared cups. The plates were incubated at 35 °C under 5% CO_2_ for 14 days. After incubation, the anti-tuberculosis activities were detected by observing the inhibition zones around the cups [28]. Each experiment was carried out in triplicate and mean value was calculated.

##### Evaluation of Anti-Tuberculous Activity by REMA Plate Assay

The REMA plate assay was carried out as described by [29]. Briefly, 100 μL of 7H9-S broth (7H9 broth + 10% OADC + 0.5% glycerol + 0.1% casitone) was dispensed in each well of a sterile flat-bottom 96-well plate, and serial twofold dilutions of the tested samples were prepared directly in the plate. One hundred microliters of inoculum, prepared as described, was added to each well. A growth control and a sterile control were also included for each isolate. Sterile water was added to all perimeter wells to avoid evaporation during the incubation. The plate was covered, sealed in a plastic bag, and incubated at 37 °C under a normal atmosphere. After 7 days of incubation, 30 μL of resazurin solution (0.02%) was added to each well, and the plate was reincubated overnight. A change in color from blue to pink indicated the growth of bacteria, and the MIC was defined as the lowest concentration of drug that prevented this change in color. Rifampicin was used as a positive control.

### 3.4. Evaluation of Cytotoxic Activity

*In vitro* cytotoxic activity of the tested samples was evaluated against normal human epithelial cell line MRC-5 as described by [30]. In such experiment, each treatment was carried out in triplicate. Drug free wells were used as negative control. Doxrubicine was used as a positive control.

### 3.5. Isolation and Structure Elucidation of the Bioactive Compound

#### 3.5.1. General

NMR experiments were performed in CDCl_3_ on a DRX-600 spectrometer (Bruker BioSpin, Billerica, MA, USA). Peak positions are reported relative to TMS. Mass spectra were measured on Acquity UPLC system (Waters, Milford, MA, USA) using a MicroTOF-Q hybrid quadrupole time-of-flight mass spectrometer (Bruker Daltonics, Billerica, MA, USA). Column chromatographic separation (CC) was performed on Diaion^®^ HP-20 (Merck, Darmstadt, Germany), silica gel 60 for column Chromatography (70–230 mesh, Merck, Darmstadt, Germany). TLC was performed on pre-coated TLC plates with silica gel 60 F_254_ (Merck, Darmstadt, Germany).

#### 3.5.2. Biologically Guided Isolation of Active Compounds

Freeze dried powder of MS-6-6 growth supernatant was dissolved in water, and fractionated on Diaion HP-20 using water, methanol/water and methanol to give fractions A-1, A-2, and A-3, respectively. The solvent was evaporated under vacuum to give fractions A-1 (30 g), A-2 (20 g) and A-3 (2 g). Antimicrobial activity of each fraction was evaluated (2 mg/mL) against the tested standard strains. The bioactive fraction (A-3) was chromatographed over silica gel column (25 × 2 cm, 50 g), using petroleum ether/ethyl acetate (1:1) as an eluent to afford active compound MY-A3. The structure of the active compound was determined by comprehensive analyses of its 1D and 2D NMR, HRESI-MS and comparison with previously reported data. Compound MY-A3 was isolated as a white amorphous powder, HR-ESIMS *m/z* 512.3406 [M+Na]^+^ (calc. for C_28_H_43_NO_6_Na) in positive mode and *m/z* 488.2874 [M−H]^−^ (calc. for C_28_H_42_NO_6_); see Table 2 for ^1^H-, ^13^C-NMR (600 MHz, 150 MHz, CDCl_3_).

### 3.6. Improvement of Antimicrobial Productivity

In such experiment, the fermentation process was done as described using different culture media (Table 4). The antimicrobial activity of the fermentation clear supernatant was evaluated as described. For quantitative determination of TP in the fermentation supernatant LC-DAD/MS analysis was carried out.

### 3.7. LC-DAD/MS Analysis of Trepenomycin and Its Metabolites in Different Media

#### 3.7.1. Apparatus and Conditions

The HPLC system consisted of an Agilent 1200 system, a solvent delivery module, a quaternary pump, an autosampler, a diode-array detector (DAD), and a column compartment (Agilent Technology, Waldbronn, Germany). The column effluent was connected to an Agilent 6320 Ion Trap HPLC-ESI-MS. The column heater was set to 25 ± 2 °C. The control of the HPLC system and data processing were performed using ChemStation (Rev. B.01.03 SR2(204), Agilent Technology, Waldbronn, Germany) and 6300 Series Trap Control version 6.2 Build No. 62.24 (Bruker Daltonik GmbH, Bremen, Germany). The analytes were separated using an Agilent Zorbax SB-C18 column (80 A°, 150 mm length × 4.6 mm, i.d., 3.5 μ) (Agilent Technologies, Palo Alto, CA, USA). The mobile phase was prepared by mixing 760 mL of 0.1% ammonia solution (25%, v/v) in water with 240 mL acetonitrile and was pumped at a flow rate of 0.5 mL/min. General MS adjustments were set as follows: capillary voltage, 3500 V; nebulizer, 35 psi; drying gas, 12 L/min; desolvation temperature, 350 °C; ion charge control (ICC) smart target, 150,000; and max accumulation time, 150 ms. The UV detector was set at 210 nm. Two MS modes were used, including the negative Auto-MSn scan mode and the single ion monitoring (SIM) mode. The Auto-MSn mode was used to help with the identification of the chemical structures of metabolites, whereas the SIM mode was applied for the quantitative analysis of the parent compound and its diasteriomer. All samples were injected applying negative single ion monitoring at m/z 488.1 for quantitative analysis. Injection volume was 5 µL.

#### 3.7.2. Preparation of *Streptomyces* Extracts from Different Media for LC-DAD/MS Analysis

Lyophilized *Streptomyces* supernatant from different media (200 mg, each) were separately extracted with 2 mL of methanol in an ultrasonic bath for 20 min. The obtained methanol extracts were centrifuged and the supernatants were passed through 0.45 HPLC membrane filters.

## 4. Conclusions 

*Streptomyces mutabilis* isolated from soil samples in Saudi Arabia produced an anti-tuberculous polyketide macrolide identified as treponemycin (TP). TP also showed a broad spectrum of activity against the Gram positive, Gram negative strains, and *Candida albicans*. Modification in growth media leads to improvement of TP productivity (150%) of *S. mutabilis*. During quantitative determination of TP titer by LC/MS after optimization, other metabolites were detected and tentatively identified based on their fragmentation pattern in order to shed light on the differences in metabolites profiling, and not only TP concentration, due to these media changes. Unfortunately, the concentration of these metabolites (T1–T4) was in nano-grams, which make their isolation impossible at this concentration level. Future work is currently underway to optimize their concentration to facilitate their isolation and assessment of activity.

## Supplementary Materials

Supplementary materials can be accessed at: http://www.mdpi.com/1420-3049/20/02/2576/s1.
