# Supplementary material for: Anti-Tuberculous Activity of Treponemycin Produced by a Streptomyces Strain MS-6-6 Isolated from Saudi Arabia
Source: Molecules. 2015 Feb 2;20(2):2576–90. doi: 10.3390/molecules20022576 (PMC6272392; doi:10.3390/molecules20022576)
Supplement: Supplementary file 1 [file molecules-20-02576-s001.pdf]

Supplementary Information

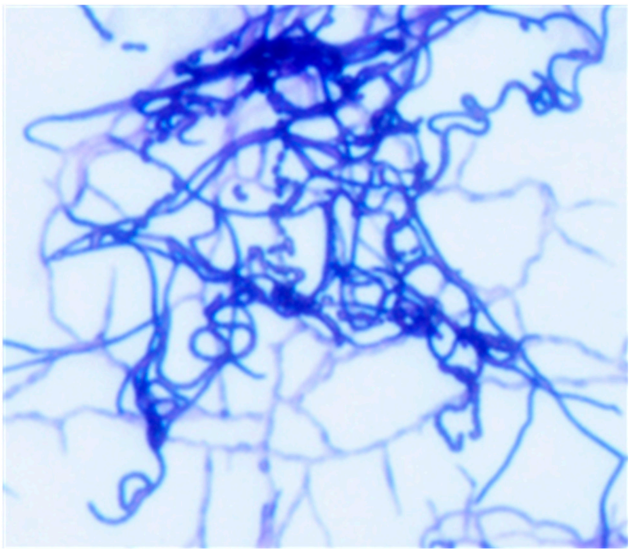

Figure S1. Photograph of *Streptomyces* strain MS-6-6 as appeared under light microscope.

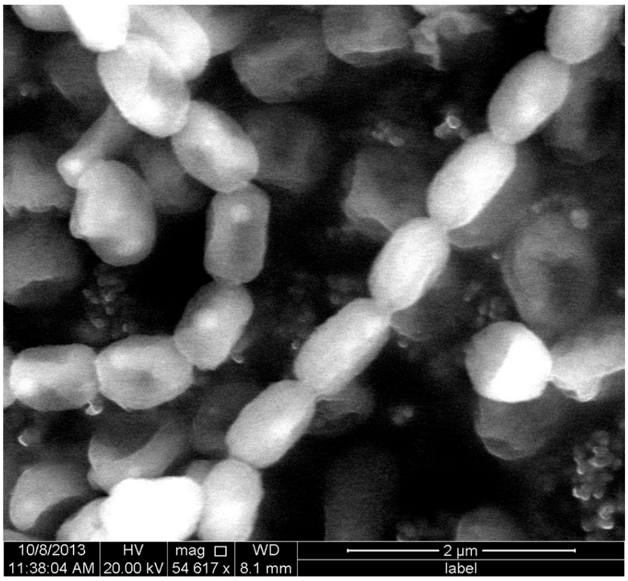

Figure S2. Electrographs of the spores of *Streptomyces* strain MS-6-6 as appeared under scanning electron microscope, 54,000×.

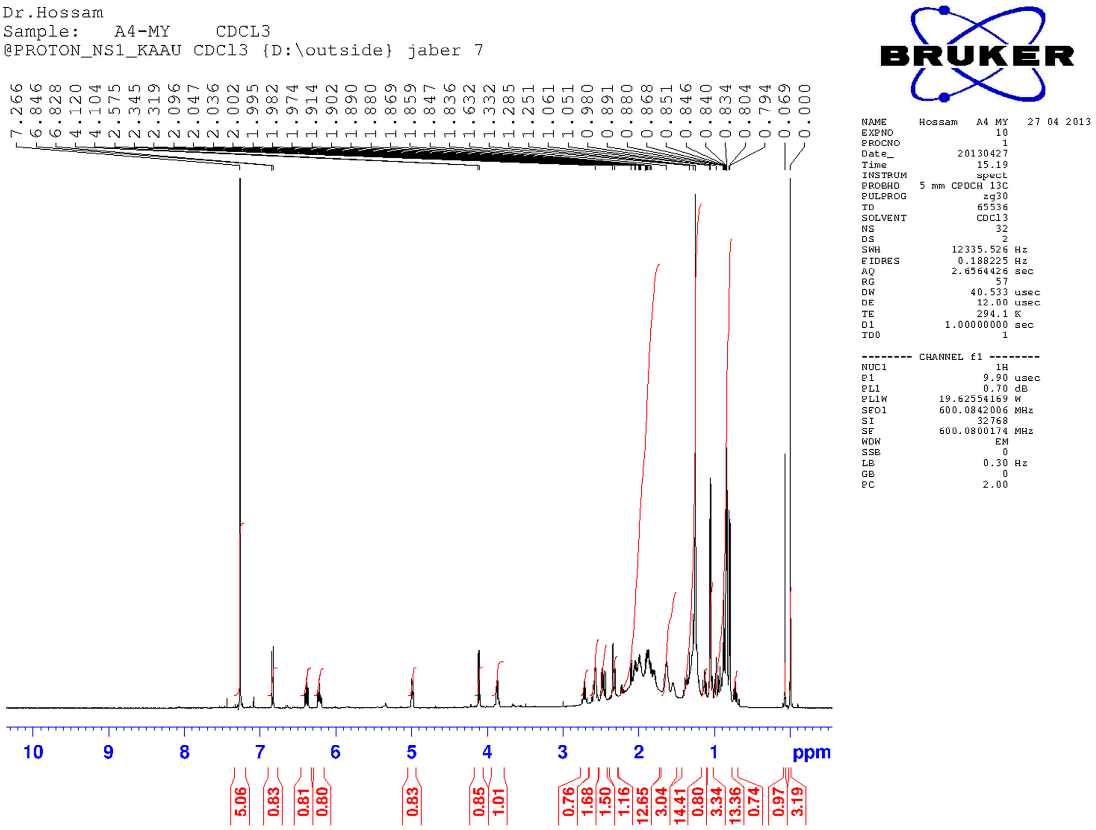

Figure S3. ^{1}H-NMR of compound (MYA-3) ($CDCl_3$).

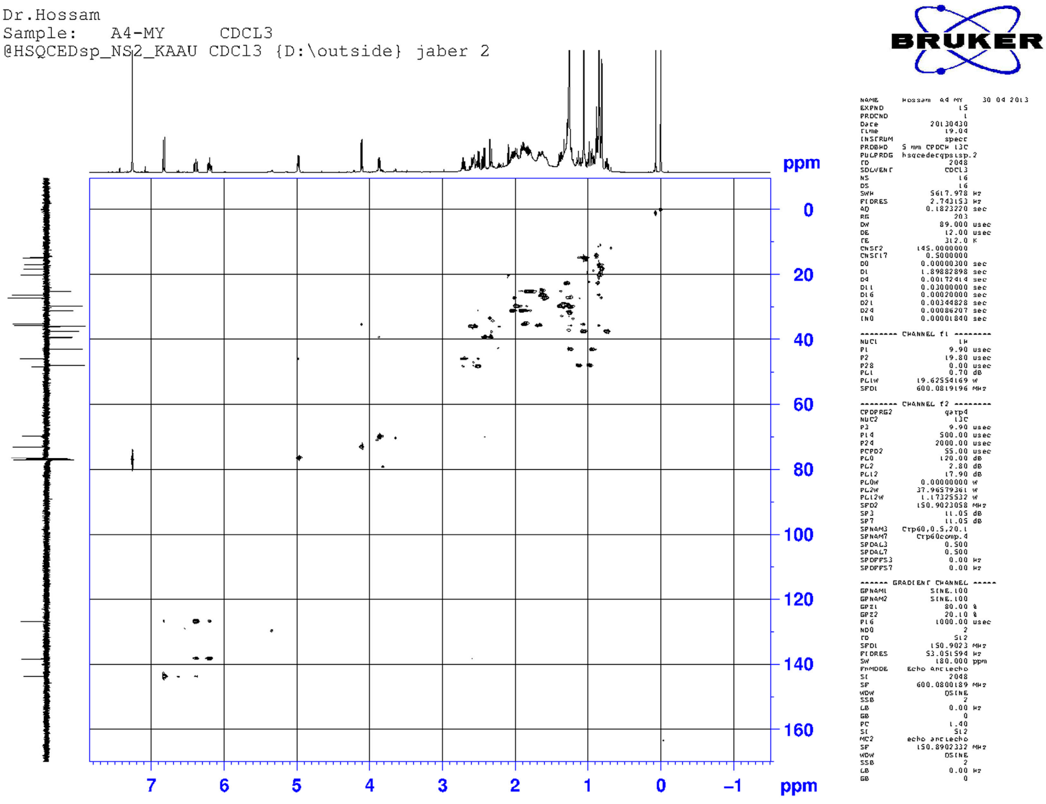

Figure S4. HSQC of compound (MYA-3) ($CDCl_3$).

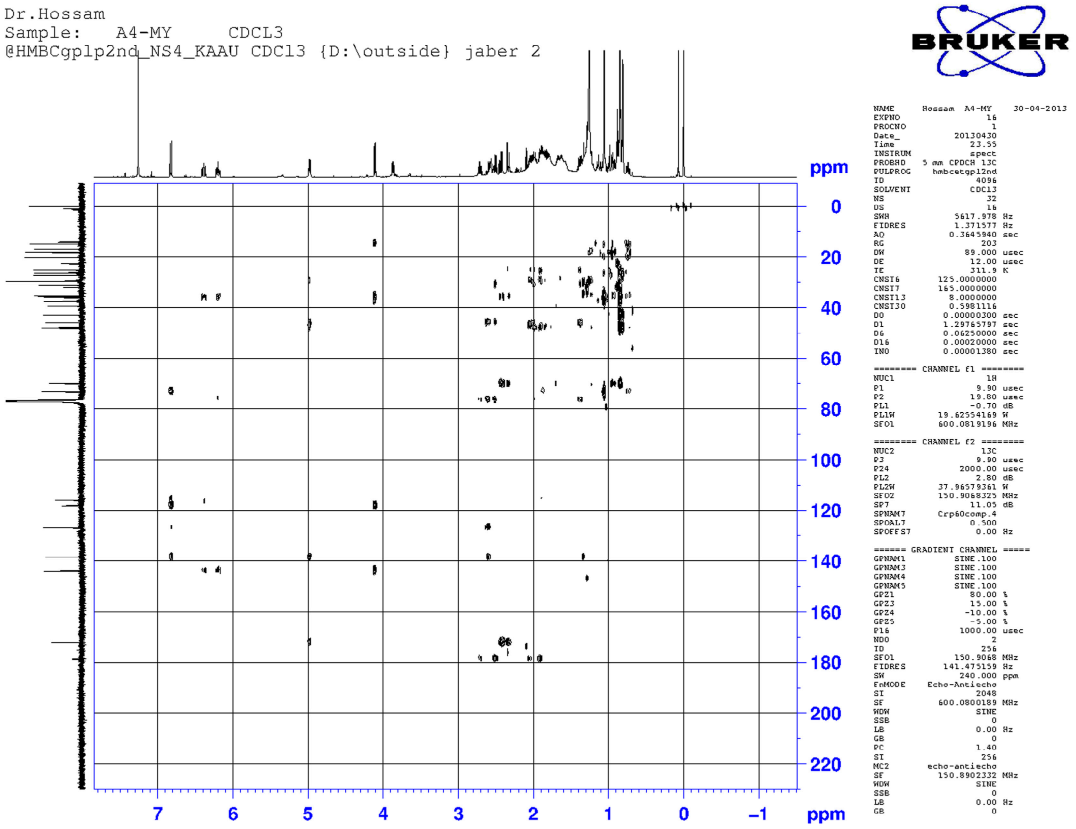

Figure S5. HMBC of compound (MYA-3) ($CDCl_3$).

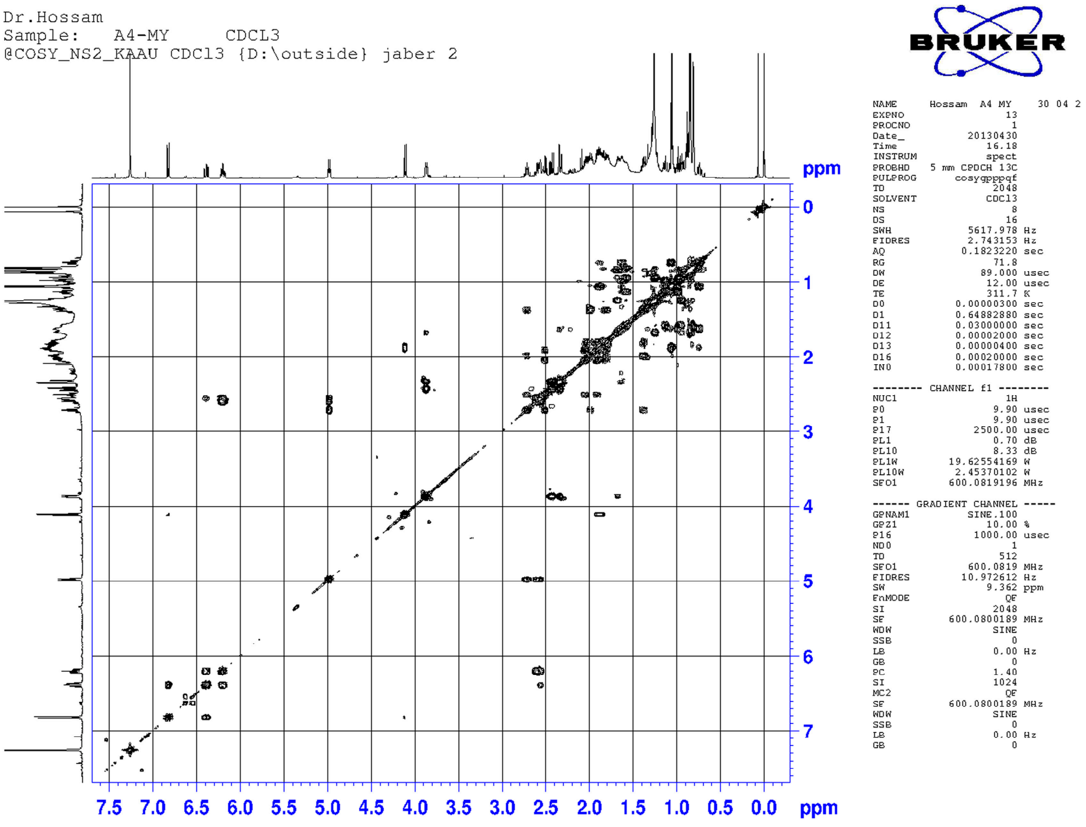

Figure S6. COSY of compound (MYA-3) ($CDCl_3$).

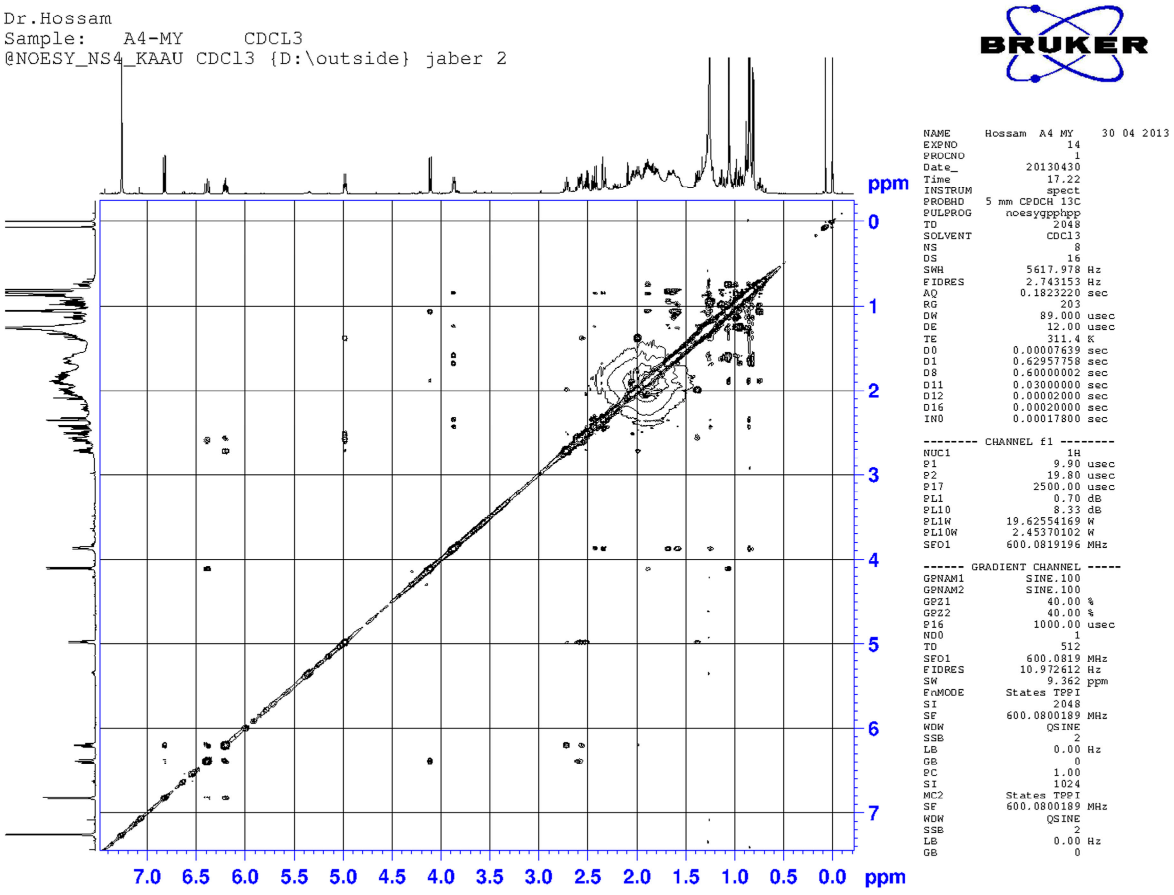

Figure S7. NOESY of compound (MYA-3) ($CDCl_3$).

Table S1. The *Streptomyces* isolates that showed different degrees of anti-tuberculous activity.

**Level of Activity (Inhibition Zones/mm)**	**Number of Isolates in Each City**					**Total**
	**Jeddah**	**Al-Medinah**	**Makkah**	**Altaif**	**Rabigh**	
Weak (≤15 mm)	39	23	14	38	4	118
Intermediate (15–20 mm)	11	6	4	6	2	29
Strong (≥20 mm)	3	2	1	1	1	8
Total	53	31	19	45	**7**	155

Table S2. Morphological and physiological properties of the Streptomyces strain MS-6-6.

Characters	Results
Spores	Spores have Rectiflexibiles spore chains, (straight to flexuous). Spore surface is smooth to slightly warty.<br>Spore mass is gray.<br>Reverse color is light brown diffusible pigments.
Growth temperature range	Growth occurred at 20, 25, 30 and 37 but no at 50 °C
Growth pH range	Growth occurred at the pH range of 5.7–8.4, optimum growth was at pH 7
Oxygen requirement	No growth occurred under anaerobic condition
Starch hydrolysis	Positive
Gelatin hydrolysis	Positive
Casein hydrolysis	Positive
Nitrate reduction	Negative
H_2S formation	Negative
Cellulose utilization	Negative
Melanin production	Negative

Table S3. Utilization of compounds as a sole source of carbon by *Streptomyces* strain MS-6-6.

Carbon Source	Results
No carbon	(−)
D-Glucose	(+)
D-Fructose	(+)
Lactose	(+)
Sucrose	(−)
Raffinose	(+/−)
D-Mannitol	(+)
meso-Inositol	(−)
L-Arabinose	(+)
Salicin	(+)
D-Galactose	(+)
Maltose	(+)
Dextrine	(+)
Cellulose	(−)
Starch	(+)

(+) = visible growth; (−) = no growth.

Table S4. Susceptibility of the Streptomyces MS-6-6 to different antibiotics.

Antibiotic	Code	Sensitivity	Antibiotic	Code	Sensitivity
Cefuroxime	CXA	R	Levofloxacin	LEV	S
Cephalothin	KF	R	Norfloxacin	NOR	S
Cefoxitin	FOX	R	Ciprofloxacin	CIP	S
Imipenem	IPM	R	Ofloxacin	OFX	S
Ceftazidim	CAZ	R	Moxifloxacin	MXF	S
Ceftriaxone	CRO	R	Nalidixic acid	NA	R
Amoxicillin clavulanic acid	AMA	R	Sulfamethoxazol/Trimethoprime	SXT	S
Oxacillin	OX	R	Doxycycline	DO	S
Piperacillin	PRL	R	Tobramycin	TOB	S
Aztreonam	ATM	R	Streptomycin	S	S
Cloxacillin	OB	R	Gentamicin	CN	S
Bacitracin	B	R	Novobiocin	NV	S
Aminocidin	AN	S	Metronidazol	MET	R
Fucidic acid	FD	R	Rifampicin	RA	R
Teicoplanin	TEC	S	Chloramphenicol	C	R

Sensitive (S) ≥ 15 mm; Resistance (R) < 15 mm.

Table S5. Antimicrobial activities of the A-1, A-2, and A-3 fractions.

Organisms	Inhibition Zones (mm)		
	A-1	A-2	A-3
Mycobacterium tuberculosis	--	14	24
Staphylococcus epidermidis	--	15	22
Streptococcus pyogenes	--	14	20
Bacillus subtilis	--	13	19
Escherichia coli	--	11	17
Clostridium perfringens	--	--	15
Brucella melitensis	--	--	16
Pseudomonas aeruginosa	--	--	15
Proteus mirabilis	--	11	16
Candida albicans	--	--	15

A-1, A-2 and A-3 are fractions collected after application of MS-6-6 growth supernatant on Diaion HP-20 using water, methanol/water and methanol, respectively.
